# Recent advances in the use of CRISPR/Cas for understanding the early development of molecular gaps in glial cells

**DOI:** 10.3389/fcell.2022.947769

**Published:** 2022-09-02

**Authors:** Carla Patricia Barragán-Álvarez, José Miguel Flores-Fernandez, Oscar R. Hernández-Pérez, Daniela Ávila-Gónzalez, Nestor Fabian Díaz, Eduardo Padilla-Camberos, Octavio Dublan-García, Leobardo Manuel Gómez-Oliván, Nestor Emmanuel Diaz-Martinez

**Affiliations:** ^1^ Laboratorio de Reprogramación Celular y Bioingeniería de Tejidos, Biotecnología Médica y Farmacéutica, Centro de Investigación y Asistencia en Tecnología y Diseño Del Estado de Jalisco, Guadalajara, Mexico; ^2^ Departamento de Investigación e Innovación, Universidad Tecnológica de Oriental, Oriental, Mexico; ^3^ Department of Biochemistry & Centre for Prions and Protein Folding Diseases, University of Alberta, Edmonton, AB, Canada; ^4^ Departamento de Fisiología y Desarrollo Celular, Instituto Nacional de Perinatología, México City, Mexico; ^5^ Laboratorio de Alimentos y Toxicología Ambiental, Facultad de Química, Universidad Autónoma Del Estado de México, Toluca, México

**Keywords:** glial cells, CRISPR/Cas, lineage tracing, gliogenesis-related genes, screening platforms, large-scale maps of cell lineage

## Abstract

Glial cells are non-neuronal elements of the nervous system (NS) and play a central role in its development, maturation, and homeostasis. Glial cell interest has increased, leading to the discovery of novel study fields. The CRISPR/Cas system has been widely employed for NS understanding. Its use to study glial cells gives crucial information about their mechanisms and role in the central nervous system (CNS) and neurodegenerative disorders. Furthermore, the increasingly accelerated discovery of genes associated with the multiple implications of glial cells could be studied and complemented with the novel screening methods of high-content and single-cell screens at the genome-scale as Perturb-Seq, CRISP-seq, and CROPseq. Besides, the emerging methods, GESTALT, and LINNAEUS, employed to generate large-scale cell lineage maps have yielded invaluable information about processes involved in neurogenesis. These advances offer new therapeutic approaches to finding critical unanswered questions about glial cells and their fundamental role in the nervous system. Furthermore, they help to better understanding the significance of glial cells and their role in developmental biology.

## Introduction

The central nervous system (CNS) is made up of a complex cell network comprised of diverse types of neurons, macroglia, and microglial cells that play a fundamental role in its proper function (Ceprian and Fulton, 2019; Nott et al., 2019; Wright-Jin and Gutmann, 2019). Glial cells can be described as progenitor cells as well as differentiated populations. Radial glial cells (RGCs) are the primary stem cells during neural development. The differentiated population involves astrocytes, oligodendrocytes, ependymal cells, Schwann cells, microglia, and among others (Arai and Lo, 2017).

While neurons have always been the protagonists of the nervous system because they are involved in synaptic interactions and electrical signaling, it was not until recently that the role of glial cells received the same attention (Wang and Gao, 2019). For many years, glial cells were considered connective tissue with the sole function of preserving nervous system junctions. However, more functions have been discovered in recent years, such as their role in neurotransmission, nutrient transport, brain functions, pathological conditions, and early development of the nervous system (Hirbec et al., 2020).

The interest in glial cell roles in neurological function has increased, focusing specifically on their dynamic interaction and involvement in neurological disorders. Moreover, recent findings open up a wide range of new opportunities for modeling early development and diseases; additionally, the discovery of biomarkers to understand pathologies causing neurodegeneration (McAvoy and Kawamata, 2019; Raikwar et al., 2019; Wang and Gao, 2019; Hidalgo-Lanussa et al., 2020; Dang et al., 2021), as well as the development of new drugs (Möller and Boddeke, 2016).

Genome engineering for modeling neuronal diseases is an emerging clinical application with a significant public health impact. However, in terms of glial cells, there is still a considerable gap in understanding origins, lineage progression, and molecular properties (Yang et al., 2022). The widely described Clustered Regularly Interspaced Short Palindromic Repeats (CRISPR)/Cas system has become a key tool for genome editing since it provides advantages such as design simplicity, considerable cost reduction, and enhanced efficiency than its analogs, Zinc Finger Nucleases (ZFN) and Transcription Activator-like Effector Nucleases (TALENs) (Moon et al., 2019). In addition, CRISPR/Cas system offers other potential clinical applications, such as gene screening or combination with orthogonal methods, thereby increasing its potential for developing new diagnostic or research tools. For instance, screening methods such as Perturb-Seq, CRISP-seq, and CROP seq, examine how diverse mutations affect specific cell types; the method Genome Editing of Synthetic Target Arrays for Lineage Tracing (GESTALT) can be combined with single-cell RNA sequencing (scRNA-seq) to find/analyze cellular lineage relationships and catalog the cell identities in different tissues; similarly, the LINeage Tracing by Nuclease-Activated Editing of Ubiquitous Sequences (LINNAEUS) is a strategy for simultaneous lineage tracing and transcriptome profiling in thousands of single cells (McKenna et al., 2016; Raj et al., 2018; Spanjaard et al., 2018; So et al., 2019; Jin et al., 2020). On the other hand, recent studies have established the basis for CRISPR/Cas9 genome editing frameworks that seem promising for neuroscience knowledge and neurological disorders treatment (Cota-Coronado et al., 2019a; Dever et al., 2019; Hirbec et al., 2020; Jin et al., 2020). Although scRNA-seq has been applied for the study of different cell phenotypes in the CNS, there are still multiple gaps in fully understanding the molecular mechanisms of glial-neuron interactions during development, as well as the role of glial cells in neuroinflammation, neurodegenerative diseases, and inherited mental disorders (Menon et al., 2019; Nott et al., 2019; Hidalgo-Lanussa et al., 2020). Therefore, this review highlights recent advances in using CRISPR technology for a better comprehension of glial cells and their role in developmental biology.

### Glial cell types and functions

Glial cells are defined as non-neuronal cells in the CNS and derive from different origins; for example, macroglia (astrocytes, oligodendrocytes, and NG2 glia) origin is the ectoderm and arise from neuroepithelial progenitor cells (NPCs); in contrast, the origin of microglia is the mesoderm, specifically from the yolk sac and its precursors are fetal macrophages (Zuchero and Barres, 2015; Arcuri et al., 2017; Yildirim et al., 2019; Patro and Patro, 2022). Glial cells are involved in nervous system regulation from development to maturation. On the other hand, they can influence nervous system plasticity and are implicated in the appearance of neurodegenerative diseases (Vallejo et al., 2019; Dietz et al., 2020). Research on glial cells began in the second half of the 19th century. However, it was not until 1919 that Rio-Hortega described for the first time the three main types of glial cells present in the CNS: astrocytes, oligodendrocytes, and microglia (Sierra et al., 2016; Sierra et al., 2019).

The nervous system development implies complex processes with extensive nuclear movements and cell migration. During early embryonic development, the neural tube emerges from the neural plate, where neuroepithelial cells (NECs) reside. These cells give rise to the radial glial cells (RGs), cornerstones of neurogenesis and gliogenesis (Arai and Lo, 2017; Bertipaglia et al., 2018). RGs nuclei exhibit a particular form of cell cycle-dependent oscillatory behavior known as interkinetic nuclear migration (INM), where the nucleus migrates within the cytoplasm basally or apically, depending on the cell cycle stage. RGs are present during most of the cortical development and divide symmetrically for self-proliferation or asymmetrically to generate neurons and glial cells (Arai and Lo, 2017; Bertipaglia et al., 2018; Lin et al., 2021).

Currently, three different types of RGs have been identified: ventricular RGs (vRGs), outer RGs (oRGs), and truncated RGs (tRGs). Yang et al., 2022 have proposed that tRGs give rise to progenitor cells of pyramidal neurons (PyN-IPCs) becoming upper layer Pyramidal neurons (PyNs). Then they produce basal multipotent intermediate progenitor cells (bMIPCs). However, there are multiple gaps related to the role of the developing cortex and the series of steps required to generate the remaining types of cells during gliogenesis (Yang et al., 2022).

**FIGURE 1:**
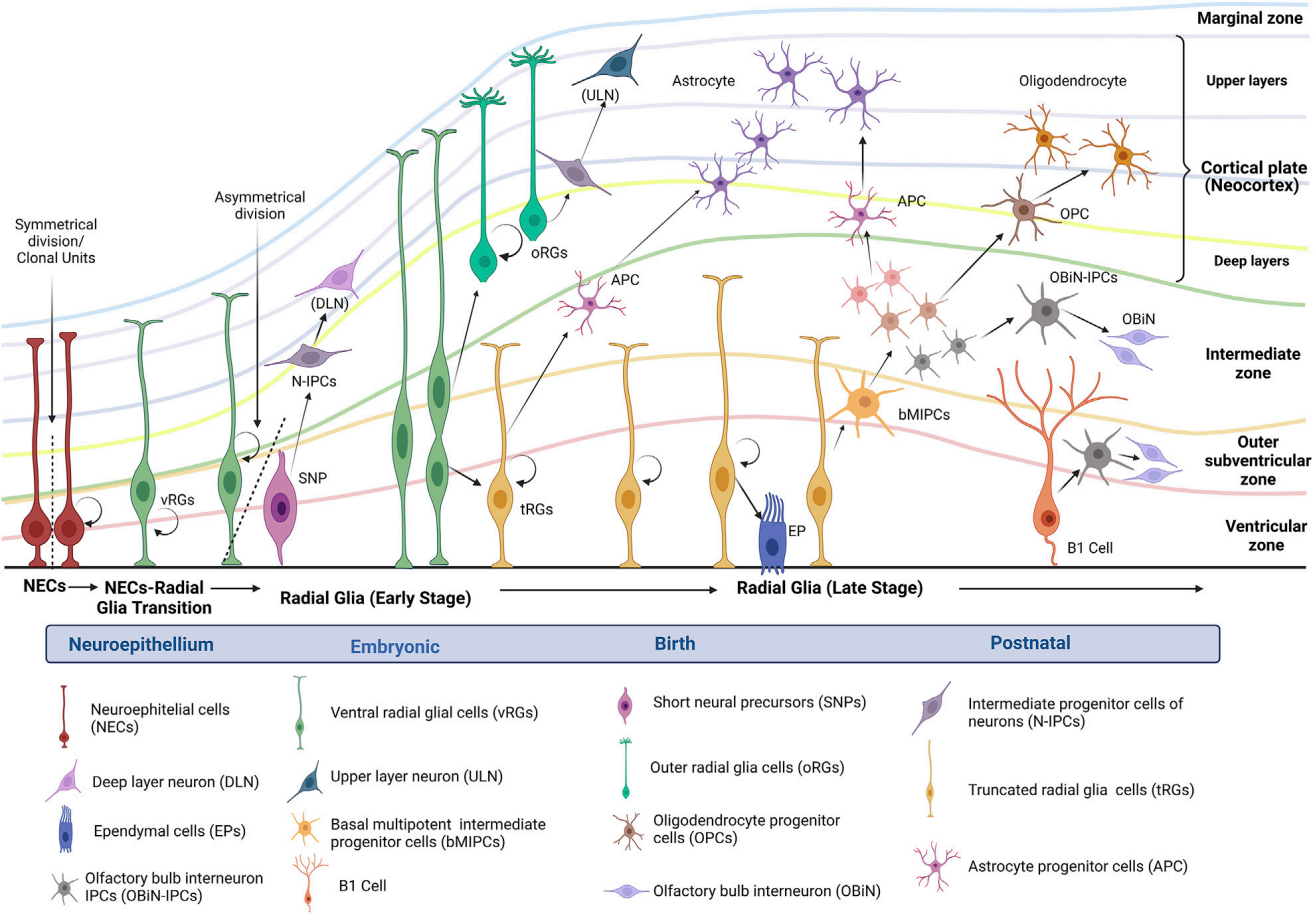
Human radial glial cell lineage progression. Figure modified of Yang et al., 2022 (Created with BioRender.com). After neural tube maturation, neuroepithelial stem cells undergo a transition to radial glia progenitors (RGs). The divisions of RGs take place in the ventricular zone. During brain development, the subventricular zone is formed as the progenitor cells delaminate from the ventricular zone. Altogether, these zones include the germinal strata that give rise to the neurons that migrate into the cortical plate and start the neocortex. Depending on the polarity in the germinal strata, RGs can be identified as ventricular RGs (vRGs) or outer RGs (oRGs). vRGs can divide symmetrically for self-renewal and asymmetrically to generate short neural precursors (SNPs) and intermediate progenitor cells of neurons (N-IPCs). N-IPCs from SNPs originate neurons that migrate to deep layers. On the other hand, some vRGs begin to detach from the apical side and transform into astrocyte progenitor cells (APC) (Kriegstein and Alvarez-Buylla, 2009; Gilbert and Barresi, 2017; Li et al., 2021). According to Yang et al., 2022, around GW15-GW16, vRGs horizontally divide into outer RGs (basal) and truncate RGs (apical); both can self-renew or differentiate into neurons of upper layers. Some truncated RGs also can transform into ependymal cells (EPs); subsequently, truncated RGs generate basal multipotent intermediate progenitor cells (bMIPCs), which can produce oligodendrocyte progenitor cells (OPCs), astrocyte progenitor cells (APC), and olfactory bulb interneuron IPCs (OBiN-IPCs). These progenitors continue their differentiation and turn into oligodendrocytes, astrocytes, and OOBiN. In the neonate, cortical truncated RGs or B1 cells continue generating neurons and oligodendrocytes and, in the first year of life, may mainly generate OBiN-IPCs (Kriegstein and Alvarez-Buylla, 2009; Arai and Lo, 2017; Yang et al., 2022).

In addition to serving as stem cells, RGs provide the scaffolding for the movement of progenitor cells and newborn neurons to superficial layers (Gilbert & Barresi, 2017). Figure 1 describes the general radial glial cell lineage progression known and proposed to date in humans.

Glial cells are also found in the peripheral nervous system (PNS) as Schwann cells, satellite glial cells, olfactory ensheathing cells, and enteric glia, whose origin is the neural crest but with at least a subset coming from the CNS that migrates to the PNS (Suter and Jaworski, 2019; Verkhratsky et al., 2019). The function of the glial cell begins at the early stages of life during brain development since glial cells facilitate neuron interactions and synaptic pruning at the final stage of brain development, as well as releasing essential gliotransmitters and cytokines during neural development (Neniskyte and Gross, 2017). In the course of embryogenesis, the microglia colonize the early embryonic neuroepithelium and give rise to the primary immune cells of the CNS (Deverman and Patterson, 2009; Neniskyte and Gross, 2017; Kim et al., 2017).

As the major components of CNS, glial cells perform multiple activities that allow homeostatic maintenance. An example is astrocytes, which modulate synaptic structure and function and promote neuronal survival. As an outcome of their interaction with blood vessels, glial cells enable nutrient intake and metabolic support; on the other hand, they can control blood flow in the brain aside from regulating the flow of cerebral spinal fluid (Zuchero and Barres, 2015; Butt and Verkhratsky, 2018; Simhal et al., 2019) Oligodendrocytes and Schwann cells are involved in the process of myelination, which is essential for neurotransmission (Kuhn et al., 2019; Verkhratsky et al., 2019) Microglia possess numerous functions and activities beyond immune surveillance in the brain; these cells can, for instance, instruct progenitor cells about cell fate decisions, establish communication with other glial cells, influence the formation of synapses and promote neurite formation, regulate neuronal function, and even ease the myelination process (Wright-Jin and Gutmann, 2019).

### Glial cells-associated neurological disorders

Glial cells are intrinsically associated in the formation or development of the nervous system since they are involved in synaptic pruning. This process is a crucial step in maturing synaptic connections during the early stages of brain development, and if key signaling pathways between glial cells and neurons do not function properly can cause several neurodevelopmental disorders like autism, schizophrenia, and epilepsy (Neniskyte and Gross, 2017; Allen and Lyons, 2018; Lehrman et al., 2018; Sellgren et al., 2019; Yanuck, 2019). Accumulated evidence suggests that excessive synaptic pruning by microglia could contribute to synapse density reduction in patients with autism and schizophrenia (Sellgren et al., 2017; Sellgren et al., 2019; Li et al., 2020; Scott-Hewitt et al., 2020). To study interactions between microglia and neural cells, Sellgren et al. (2017) validated a model system combining reprogrammed microglia-like cells with neural progenitor cells (NPCs); their results showed the ability of microglia-like cells to engulf synaptosomes and NPCs *in vitro*; moreover, they reported risk schizophrenia variants in the human complement component 4 locus causing an excessive neuronal complement deposition by C4A, a factor associated with increased microglial synapse engulfment (Sellgren et al., 2017; Sellgren et al., 2019; Li et al., 2020; Scott-Hewitt et al., 2020).

It has been reported that microglial complement receptor CR3/Mac1 and triggering receptor expressed on myeloid cells 2 (TREM2) contribute to synaptic pruning (Qin et al., 2022). Scott-Hewitt et al. (2020) proposed that the recognition of exposed phosphatidylserine in neurons is crucial for microglial-mediated pruning, and some possible candidates as synapse pruning mediators are the isoform of adhesion G protein-coupled receptor (ADGRG1/GPR56) and TREM2; however, the molecular mechanisms involved in microglia target neuron selection are unknown (Li et al., 2020; Scott-Hewitt et al., 2020; Qin et al., 2022). On the other hand, it should be noted that TREM2 and the complement cascade have been associated with the progression of Alzheimer’s disease (AD), as mouse models have shown the importance of TREM2 for the microglial phagocytosis response in amyloid seeds (Smidt et al., 2007; Flores-Fernández et al., 2018; Parhizkar et al., 2019; Ewers et al., 2020; Meilandt et al., 2020; Scott-Hewitt et al., 2020; Yang et al., 2020). The increased presence of soluble TREM2 (sTREM2) in cerebrospinal fluid (CSF) has also been associated with an attenuated decline in memory and cognition among patients with mild cognitive impairment (MCI) and AD (Ewers et al., 2019; Suárez-Calvet et al., 2019). In addition to TREM2, genome-wide association studies (GWAS) have identified other important AD and Parkinson’s disease (PD) risk genes expressed or associated with reactivity in glial cells, such as the transmembrane protein CD33, box-dependent-interacting protein 1 (BIN1), complement receptor 1 (CR1), apolipoprotein E (ApoE), GBA1, and stearoyl-CoA-desaturase (SCD) (Park et al., 2018; Bartels et al., 2020).

Similar to AD, a recent study reported increased levels of sTREM2 in PD patients, proposing sTREM2 as a potential biomarker in both conditions. Furthermore, current evidence suggests astroglial cells as the primary source of inflammatory mediators in the brain, as well as the microglia response to pro-inflammatory signals of mast cells, where glia maturation factor (GMF) could have a central role during neuroinflammation. GMF is a growth and differentiation factor majorly expressed in CNS; it has been indicated as a pro-inflammatory protein playing a central role in neuroinflammatory and neurodegenerative diseases such as AD, PD, and multiple sclerosis (MS) (Kempuraj et al., 2018a; Kempuraj et al., 2018b; Fan et al., 2018; Raikwar et al., 2019). To date, the exact mechanism by which GMF acts on diseases is unknown; however, there is considerable evidence that helps to understand its relationship with neuroinflammation and neurodegeneration (Fan et al., 2018; Yin et al., 2018; Ramaswamy et al., 2019; Lee et al., 2021). Some reports indicate that GMF is involved in the secretion of granulocyte-macrophage colony-stimulating factor (GM-CSF); overexpression of GM-CSF may lead to the activation of microglia and the secretion of TNF-α, IL-1β, and MIP-1 β triggering an inflammatory process. In addition, GMF can activate mast cells which also release inflammatory mediators (Kempuraj et al., 2018b; Lee et al., 2021).

Moreover, GMF is involved in oxidative stress signaling. It has been reported that GMF is closely related to the dysregulation of copper-zinc superoxide dismutase (Cu-Zn SOD) and catalase and glutathione peroxidase (GPX), enhancing neurotoxicity through oxidative stress (Fan et al., 2018; Selvakumar et al., 2018). Microglia activation leads to reactive oxygen species (ROS) production, which can exacerbate oxidative stress, causing neuroinflammation and cell death (Fan et al., 2018; Selvakumar et al., 2018). On the other hand, some studies have demonstrated that in GMF-KO neurons or glial cells, the activation and release of chemokine (C-C motif) ligand 2 (CCL2) is reduced, and they are more tolerant of 1-methyl-4-phenylpyridinium (MPP+) toxicity. CCL2 is expressed in glia, neurons, and mast cells; its relevance resides in its role in PD. Up-regulation of this chemoattractant can lead to microglia over-activation leading to neuron damage and neuroinflammation. Furthermore, CCL2 released from brain cells and mast cells could be involved in infiltrating other inflammatory cells into the substantia nigra, potentiating the damage (Kempuraj et al., 2018a; Kempuraj et al., 2018b; Shen et al., 2019).

Glial cells play an essential role in the development and progression of other neurological disorders, such as amyotrophic lateral sclerosis (ALS), Fragile X Syndrome (FXS), and brain tumors (Kempuraj et al., 2018a; McAvoy and Kawamata, 2019; Raikwar et al., 2019; Selvakumar et al., 2019; Wright-Jin and Gutmann, 2019; Wilson et al., 2020) ALS is characterized by motor neuron degeneration and gliosis. New reports has been described that aberrant glial cells with highly proliferative and neurotoxic properties promotes the disease progression (Martínez-Palma et al., 2019; Filipi et al., 2020). The FXS disorder is caused by the loss of Fragile X Mental Retardation Protein (FMRP) that triggers alterations in glial cells, as demonstrated in FMRP knockout (KO) mice models where decreased hippocampal and neocortical circuitry synapses associated with astrocytes were observed (Simhal et al., 2019). On the other hand, the most common primary malignant brain tumor, glioblastoma, can modulate glial cells to regulate the microenvironment surrounding the tumor by multiple intercellular communication pathways. This tumor possesses a high infiltrative growth pattern since its boundary is composed of tumor cells, immune cells, and reactive glial cells. Several studies have demonstrated that microglial cells and astrocytes are essential for tumor progression. However, many unanswered questions still need to be clarified to improve understanding of the crosstalk role between glioblastoma and glial cells (Oliveira et al., 2017; Yekula et al., 2019; Belykh et al., 2020).

### Advances and new CRISPR/Cas applications

#### CRISPR/Cas system enhancement and common uses

The CRISPR/Cas systems are considered the adaptive immune prokaryote machinery against bacteriophages and mobile genetic elements; their applications have gone beyond genome editing in a wide variety of organisms by enhancing and renewing biotechnological tools through the specific DNA-binding ability of Cas to perform transcriptional control, modulate epigenetic modifications, live-cell imaging studies, identification of gene targets or gene signatures, cell lineage mapping, and diagnostic platforms, among other, uses (McKenna et al., 2016; Murugan et al., 2017; Duan et al., 2019; Kellner et al., 2019; Manghwar et al., 2019; Pickar-Oliver and Gersbach, 2019). The range of Cas9 variants has increased since the first use of the well-characterized *Streptococcus pyogenes* Cas9 (SpCas9). As a result of current research, we know more about new CRISPR–Cas systems than ever before, and the last classification considered two classes, six types, and 33 subtypes. Nonetheless, there is a huge reservoir of unknown CRISPR/Cas systems that could have enormous potential. Cas9 recognition depends on base pairing between the target sequence and the single-guide RNA (sgRNA) and the presence of an adjacent protospacer motif (PAM) sequence flanking the target site (Murugan et al., 2017; Manghwar et al., 2019; Makarova et al., 2020).

CRISPR/Cas9 systems are the most widely known and used. However, they have limitations, such as their large protein size, *in vivo* restrictions to optimal viral delivery, limited PAM sites, presence of off-target mutations, and low homology-directed repair (HDR) efficiency. As a way of overcoming these limitations, several studies have explored the natural diversity of the CRISPR/Cas9 system, finding suitable variants, such as the CjCas9 isolated from *Campylobacter jejuni*, which boast several benefits such as lower protein size compared to other Cas9 orthologues, having major specificity, including its ability efficient to modify the genome both *in vitro* and *in vivo* (Edraki et al., 2019; Hua, Tao, Han, Wang, & Zhu, 2019; E. Kim et al., 2017; Kleinstiver et al., 2016; Manghwar et al., 2019). Furthermore, the *Neisseria meningitides* Cas9 variants (Nme1Cas9 and Nme2Cas9) have emerged as other options for an all-in-one delivery method because their compact size can be packet in an adeno-associated virus (AAV) with a guide RNA targeting for *in vivo* applications. Additionally, Nme2Cas9 can recognize a simple dinucleotide PAM (N4CC), providing a higher target site density of genomic sites with minimal or null off-target mutagenesis (Amrani et al., 2018; Edraki et al., 2019). PAM interaction is one of the significant restrictions of Cas9 recognition since it can be challenging to generate precise genome editing if it depends on a specific PAM; a clear example is HDR, where efficiency is improved when the double-stranded breaks (DSBs) are made between 10 and 20 base pairs of the desired target (Kleinstiver et al., 2016). According to a recent study involving 79 Cas9 proteins, 50 different PAM sequences can be recognized. Most orthologs can recognize a PAM greater than 2 bp; orthologs that recognize a PAM of ≥3 bp are likely to provide a major degree of specificity. However, it is essential to be careful with a complex PAM since access will also be more restricted (Hua et al., 2019; Gasiunas et al., 2020).

The Cas9 PAM interaction domain can be engineered to recognize multiple sequence motifs due to its extraordinary flexibility. Currently, the two widely used Cas9 orthologs, *Staphylococcus aureus* Cas9 (SaCas9) and SpCas9, have been modified to have different specificity and recognize new PAM sequences; some of them are shown in Table 1. For example, xCas9 and SpCas9-NG are two newly engineered SpCas9 variants that can identify more relaxed NG PAMs (Hu et al., 2018; Hua et al., 2019; Karvelis et al., 2019; Gasiunas et al., 2020). Altogether, these new evolved variants enable targeting multiple PAM sequences and making approachable genomic sites that were previously inaccessible (Miller et al., 2020). Cas9 recognizes GC-rich PAM sequences, but Cas12a (or Cpf1) and Cas12b (or C2c1) belonging to class 2 type V of the CRISPR/Cas system offer a new option for genome engineering by recognizing AT-rich PAM sequences. Furthermore, the newly described anti-CRISPR proteins AcrIIA2, AcrIIA4, and AcrIIC2Nme with Cas inhibition effect could be used as genetically encodable “off-switch” tools for Cas9 activity (Yang and Patel, 2017; Hua et al., 2019; Sun et al., 2019; Thavalingam et al., 2019). More recently, Cas 13 protein has been described as a nuclease capable of targeting and cleaving single-stranded RNA molecules (Shmakov et al., 2015; Abudayyeh et al., 2016; Wolter and Puchta, 2018). It must also be noted that these proteins possess two enzymatically distinct RNase activities since they can cleave the pre-crRNA array to form mature Cas13-crRNA and an RNA target complementary to the crRNA. These qualities make Cas 13 proteins optimal for RNA interference assays and potential diagnostic and treatment tools for viral diseases (Tambe et al., 2018; Fricke et al., 2020; Yin et al., 2020; Dang et al., 2021).

**TABLE 1:** Examples of SpCas9 and SaCas9 PAM engineered variants.

Variants	Origin Specie	PAM	Tested organisms	Advantages	Strategy for engineered	References
VRER-Cas9	*Streptococcus pyogenes*	NGCG and NGA	Human cells (U2OS cells)	Similar (or better) genome-wide specificities compared to wild-type SpCas9	Bacterial selection-based directed evolution, and combinatorial design	Kleinstiver et al. (2016)
VQR-Cas9						
KKH SaCas9	*Staphylococcus aureus*	NNNRRT	Human cells (U2OS cells)	Comparable or slightly lower levels of mutagenesis compared with SaCas9 wild type	Molecular evolution	Kleinstiver et al. (2016)
SaCas9-HF	*Staphylococcus aureus*	NNNRRT	Human retinal pigmented epithelium cell line	Reduced off-target effects than SaCas9	Site-directed mutagenesis	Tan et al. (2019)
SpCas9-NRRH	*Streptococcus pyogenes*	NRRH, NRCH and NRTH	Human cells (HEK293T cells)	Higher on-target activity and similar or fewer numbers of detected off-target sites compared to SpCas9	Phage-assisted evolution	Miller et al. (2020)
SpCas9-NRCH						
SpCas9-NRTH						
xCas9	*Streptococcus pyogenes*	GAT and NG	Rice	Higher specificity than SpCas9	Codon-optimization by PCR	Hua et al. (2019)
SpCas9-NG						

Since CRISPR/Cas is recognized as a natural genome editing tool, targeting a DNA/RNA sequence to monitor, break down, or replace, reverting the versions of diseased genes to a healthy version of the gene. This technology has been widely used to treat human genetic disorders, diagnose human diseases, and help detect diseases early. Beyond those usages, it has been used for creating animal genetic models to assist in the understanding of human genetic diseases; however, it is potential usage for understanding the early development of molecular gaps in glial cells, gene editing of human neural stem cells (NSC), and RGs have been poorly studied (Abdelnour, Xie, Hassanin, Zuo, & Lu, 2021; Cota-Coronado et al., 2019b, Ramírez-Rodríguez, et al., 2019).

### CRISPR/Cas is strategy to study glial cells

#### CRISPR applications for the study of glial cells-associated neurological disorders

Due to its enormous potential for multiple applications, the CRISPR/Cas system has been widely used to study neurodegenerative diseases. The system has assisted in understanding the molecular processes involved in the dynamic interaction between CNS cells and neurodegeneration. Genome examination with the CRISPR/Cas system leads to the discovery of potential markers in the early stages of neurodegeneration; on the other hand, epigenetic and gene editing can drive precision-targeted regenerative therapies (Cota-Coronado et al., 2019a; Raikwar et al., 2019; Kampmann, 2020; Ruetz et al., 2021).

The mechanisms and role of glial cells in the CNS and in treating neurodegenerative disorders could be understood using CRISPR/Cas. As mentioned above, critical glial genes are involved in neurological pathologies like the TREM2 gene. An innovative study employing CRISPR/Cas in an induced pluripotent stem cell (iPSC) model differentiated to microglia, demonstrated that TREM2-KO reduces microglial survival, alters its phagocytosis function, and results in an impaired response to beta-amyloid plaques, thus revealing possible mechanisms that may have an essential role in AD progression (McQuade et al., 2020). However, other mechanisms related to TREM2 signaling must be elucidated as possible participation in synaptic pruning. A dual role has been proposed since TREM2 may contribute to plaque containment and clearance or aberrant synaptic loss (Scott-Hewitt et al., 2020). Hence, attention to TREM2 has been remarked on as a therapeutic target for its ability to modulate the microglial function and as a biomarker in the early stages of AD (Ewers et al., 2019; Ewers et al., 2020). Another target gene studied using the CRISPR/Cas9 method is GMF since it possesses a proinflammatory effect and is significantly upregulated in different zones of the AD brain. GMF expression is predominant in the reactive glial cells surrounding the amyloid plaques (APs). Raikwar et al. (2019) inhibited GMF expression in the microglial cell line BV2 by transducing them with lentiviral vectors that expressed SpCas9 and GMF-sgRNAs; they observed a reduction of microglial activation and suggested that *in vivo* GMF gene editing could be considered as a novel AD therapy (Raikwar et al., 2019). In addition, it has been demonstrated that GMF-KO in microglial cells ameliorates microgliosis as a consequence of improved mitochondrial dynamics and oxidative stress. In acordance with this, Selvakumar et al. (2019) shown that oxidative stress generated for microglial cells is associated with PD.

#### CRISPR approaches in the early development of glial cells

The CRISPR/Cas system allows the study of genes associated with neurodegenerative diseases and offers therapeutic approaches such as gene editing of NSC and RGs. As mentioned above, RGs are the primary stem cells during neural development and play a crucial role in neurogenesis and gliogenesis. However, it is necessary to extend our understanding of these cells' origins, lineage relationships, the timing of differentiation, and molecular properties (Li et al., 2021; Yang et al., 2022). The electroporation is a common technique employed for a rapid and efficient *in vivo* delivery of CRISPR/Cas9 system components into neural stem cells of the embryonic neocortex by *in utero* electroporation and/or microinjection into single neural stem cells in neocortical tissue, to investigate the function of specific genes during embryonic brain development (Kalebic et al., 2016). A similar approach known as Easi-CRISPR (Efficient additions with ssDNA inserts-CRISPR) has recently been adapted to target the developing brain by electroporating neurons with ribonucleoprotein complexes (Cas9 + crRNA + tracrRNA) which allows the editing of neural clonal lineages to be selective. Similar Breasi-CRISPR can reveal protein-protein interactions in the developing cortex, tagged proteins by immunoblot analysis in a single cortex just 2 days after electroporation and, by immunohistochemistry in 24 h. Using these techniques, we can elucidate protein-protein interactions. Thus, we can analyze the role played by endogenous proteins during early brain development (Meyerink et al., 2022).

A recent study of integrating analysis of single-cell RNA-Seq datasets from human fetal brain samples concluded that “the developmental origins of human cortical glial cells are similar to that in the mouse cortex”(Yang et al., 2022). With these data, Yang et al. (2022) could establish a general model of RGs lineage progression (Figure 1) and the molecular identity of tRGs, which express many hallmarks of cells in the astrocyte lineage. Some molecular markers identified appear to have a central role in specific progenitors, such as epidermal growth factor receptor (EGFR) expressed in tRGs but not in vRGs or oRGs. In addition, tRGs expressing EGFR give rise to PyN-IPCs and bMIPCs positives at this marker, too (Yang et al., 2022). Previous studies have revealed that EGF is a vital mitogen to enhance oligodendroglial development (Yang et al., 2016; Yang et al., 2017). More recently, some findings provided strong evidence that EGF facilitates the transdifferentiation of astrocytes to oligodendrocytes and that the EGF-EGFR-Erk1/2 pathway could be essential in this process (Liu et al., 2022). Furthermore, markers such as HOPX, FAM107A, TNC, and LIFR have been identified in the three RGs (vRGs, oRGs, and tRGs) (Yang et al., 2022).

Understanding processes involved in gliogenesis can help define and manipulate specific subsets of neurons and glial cells, as shown in recent research where an atlas was generated from a developing zebrafish brain employing the method GESTALT. The model encompasses 12 stages of the diversification of neurons and progenitors from embryo to larva and has shown differences with other species in neurogenic programs of CNS, as well as between zebrafish and mammalian neurogenesis. For example, in zebrafish, radial glial cells persist into adulthood and contribute to neurogenesis, in contrast to mammalians, where a shift from neuronal to glial programs exists. Based on the optimized GESTALT method, cell lineage trees can be constructed and adapted for barcoding lineages on specific development windows corresponding to different branches of the specification trees or tag Campo’s interest populations (Raj et al., 2020).

#### Gliogenesis-related genes of interest

Some genes of interest still need to be studied in more detail using the techniques described in this review to determine their essential role in gliogenesis. For instance, since quite a while ago; it has been known that in different animal models, such as lampreys, Nkx2.2, PDGFR, and SoxE (the lamprey Sox10 ortholog) genes might be involved in gliogenesis (Yuan et al., 2018), but it wasn’t until the CRISPR/Cas9 gene was used in the year 2021 that it was determined SOX10 plays similar roles, but not the same ones, in the development, migration, and differentiation of the neural crest. SOX10 is essential in human neural crest development for the transition of premigratory cells to migrating neural crest, it is vital for neural crest survival, and it is required for Schwann cell development as well (Lai et al., 2021). Similarly, the Olig1 and Olig2 genes in animal models have been suggested as necessary and sufficient for oligodendrocyte precursor development in the brain of Olig2 gene null mice, but Olig1 is insufficient for the formation of motor neurons or oligodendrocytes in the embryonic spinal cord in the absence of Olig2. However, both genes have not been studied yet using technologies described in this review to elucidate their roles in the RGs, which is extremely important since these genes are located on chromosome 21 and could be associated with Down syndrome (Lu et al., 2002).

Furthermore, RGs can be studied in tissue organoid models such as a 3D cortical spheroid differentiation, a recent *in vitro* model in wich mTORC1 hyperactivation was induced, resulted in greater production of glial-lineage cells, which include astrocytes. In contrast, mTORC1 suppression strongly promoted neurogenesis and impaired gliogenesis, meaning mTORC1 is required for normal gliogenesis. Still, more studies are needed to uncover its central role in the RGs (Blair et al., 2018). In addition to this, considering studying the RGs shape is another essential factor to perform its proper function, that is why further morphological studies by technologies using CRISPR/Cas need it; for example, in one study, Pax2a gene mutants of the zebrafish resulted in defects in many aspects of the Müller glial cell morphology (Charlton-Perkins et al., 2019)**.** Furthermore, 3D brain organoid technology allows for studying human microglia functions (Cakir et al., 2022). However, most of the methods employed for brain organoid generation are based on the neuroectoderm differentiation of pluripotent stem cells. These methods hinder mesodermal lineage cell obtention and, therefore, microglia emerging (Ormel et al., 2018; Fagerlund et al., 2021; Cakir et al., 2022). Currently, some models have been developed for functional microglia representing as described in the recent work of Cakir et al. (2022) that generated microglia-containing human cortical organoids (macOS) using PU.1-induction. They proposed mhCOs as a novel platform to study the microglia-specific function; to exemplify it, they performed a pooled CRISPRi screening of AD-related genes in microglia; their findings suggested an association with dysregulation of cholesterol metabolism *via* the Sorl1 gene (Cakir et al., 2022).

It is worth mentioning that in a human neural stem cell model of astrocyte pathology, other genes of interest were found upregulated. They were associated with neurological system processes (CHRNA1, CRYZ, EYA1, NPY, PCDHB5), synapse organization, biogenesis (CHRNA1, PCDHB5) and synaptic transmission (CHRNA1, NPY, PCDHB5), founding ANXA2 gen significantly expressed in the pathology (Hallmann et al., 2017). However, its primary function is still a gap open to elucidation.

### CRISPR screening platforms, panels, and large-scale maps of cell lineage

The latest advances in pooled screening provide a powerful approach to illustrating gene function and association in a biological process, disease, or disorder. These kinds of studies are challenging due to the enormous list of genes at the genome-scale. In some cases, pooled screening has been delimited to phenotypic average properties of a population by considering only a few exogenous reporters or effects on cell viability, thus limiting the understanding of genetic perturbations of impact or the distinction between different perturbations with similar responses (Adamson et al., 2016; de Groot et al., 2018; Parekh et al., 2018; Jin et al., 2020).

**FIGURE 2:**
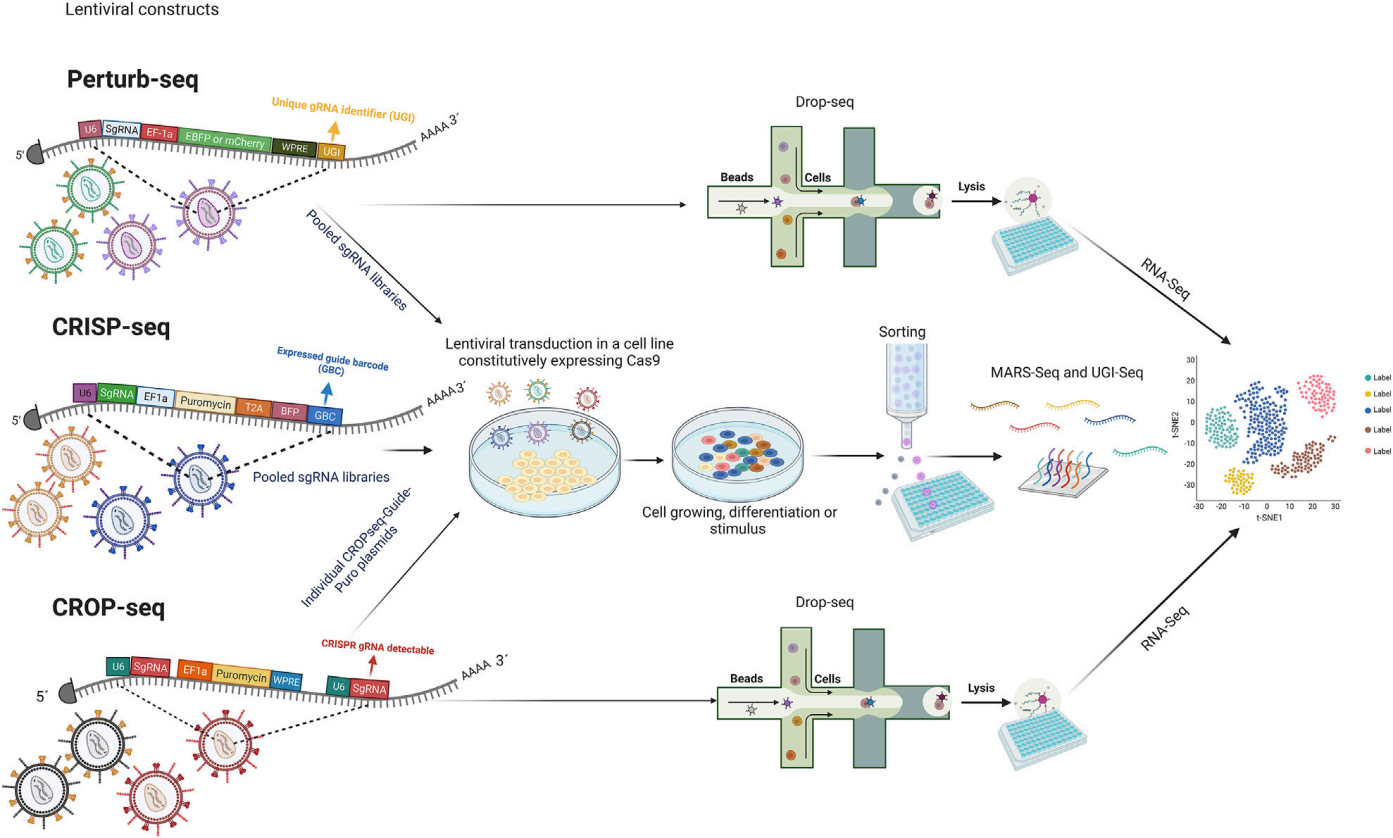
A general methodology of CRISPR screening platforms. “Figure created with BioRender.com.”Lentiviral backbone constructed in Perturb-Seq, CRISP-seq, and CROPseq share elements like the hU6 promoter, sgRNA, EF1a promoter, and some selective and reporter markers. Perturb-Seq and CRISP-seq employ barcodes for single-cell CRISPR screening, while CROP-seq reads the sgRNA directly. Lentiviral transduction is performed using pooled sgRNA in the case of Perturb-Seq and CRISP-seq; in contrast, CROPseq delivery is performed individually. After growth, differentiation, or stimulation depending on the subject, Perturb-Seq and CROP-seq perform a single-cell screening employing printed droplet microfluidics. For CRISP-seq selection, cell sorting is used. All the methods consider RNA-Seq. Human U6 (hU6); Single-guide RNA (sgRNA); Elongation factor 1-alpha (EF1a); Hepatitis post-transcriptional regulatory element (WPRE); The small 2A peptide sequences (T2A); Blue Fluorescent Protein (BFP); Unique gRNA identifier (UGI); Expressed guide barcode (GBC); RNA sequencing (RNA-Seq).

Facing these challenges, CRISPR screens have emerged, enabling a new efficient perturbation tool with multiple applications. There are two major kinds of CRISPR screens, the pooled and the arrayed. A pooled CRISPR screen typically involves a library that is introduced in bulk into a single or a group of cells under a specific treatment that leads to selecting cells whose perturbations confers a particular advantage. In contrast, arrayed CRISPR screens separate perturbations throughout the screen for a more controlled study (Bock et al., 2022). In the next few years, novel screening methods with high-content and single-cell screening at the genome-scale will be necessary. Currently, some methods have been described: Perturb-Seq, CRISP-seq, and CROPseq; their base resides on CRISPR knockout or knockdown screening in combination with single-cell-based RNA-seq, doing possible research at a single-cell level and with the projection of the study of a large-scale gene perturbation (Adamson et al., 2016; Dixit et al., 2016; Jaitin et al., 2016; Datlinger et al., 2017; Parekh et al., 2018; Duan et al., 2019; Schraivogel et al., 2020). The review work of Bock et al. (2022) describes concepts of CRISPR screening, experimental design, and applications extensively. The major difference between CROP-seq compared to Perturb-seq and CRISP-seq is that guide RNA is directly read, simplifying the single-cell CRISPR screening with large guide RNA libraries (Datlinger et al., 2017). The general mechanism through which these methodologies work can be seen in Figure 2. In addition to these approaches, the pooled genetic screens based on the CRISPR interference (CRISPRi) system allow the study of a wide range of genetic perturbations and mutagenesis for identifying gene function and gene-phenotype interactions (Schuster et al., 2019).

Like current research on single glial gene function, large gene panels in the neurobiological context are arising. Jin et al. (2020) developed a scalable genetic screen approach applying *in vivo* Perturb-Seq for the study of 35 loss-of-function risk genes for autism spectrum disorder (ASD), and they identified specific cell gene signatures in both neuronal and glial cells that are affected by genetic perturbations (Jin et al., 2020). A recent study performed with the CRISPR/Cas9 platform for gene targeting at multiple loci of NSCs for galactosylceramidase (GALC) overexpressing, a vital enzyme whose loss is associated with the death of myelin-producing oligodendrocytes and Schwann cells, gene editing allowed the reestablishment of GALC activity when edited NSCs were transplanted into oligodendrocyte mutant shiverer-immunodeficient mice (Dever et al., 2019). On the other hand, Lalli et al. (2020) studied a set of 13 ASD-associated genes by coupling pooled dCas9-based transcriptional repression to single-cell RNA-seq in a human model of neuron differentiation; their findings evidenced unique and overlapping consequences on transcriptional networks and pathways of these genes on cell-cycle, and five of them (ADNP, ARID1B, ASH1L, CHD2, and DYRK1A) were identified as the cause of delay neuron differentiation. Additionally, they predicted that PTEN (phosphatase and tensin homolog) repression could have a positive effect by increasing proliferation and neuron projection (Lalli et al., 2020).

**FIGURE 3:**
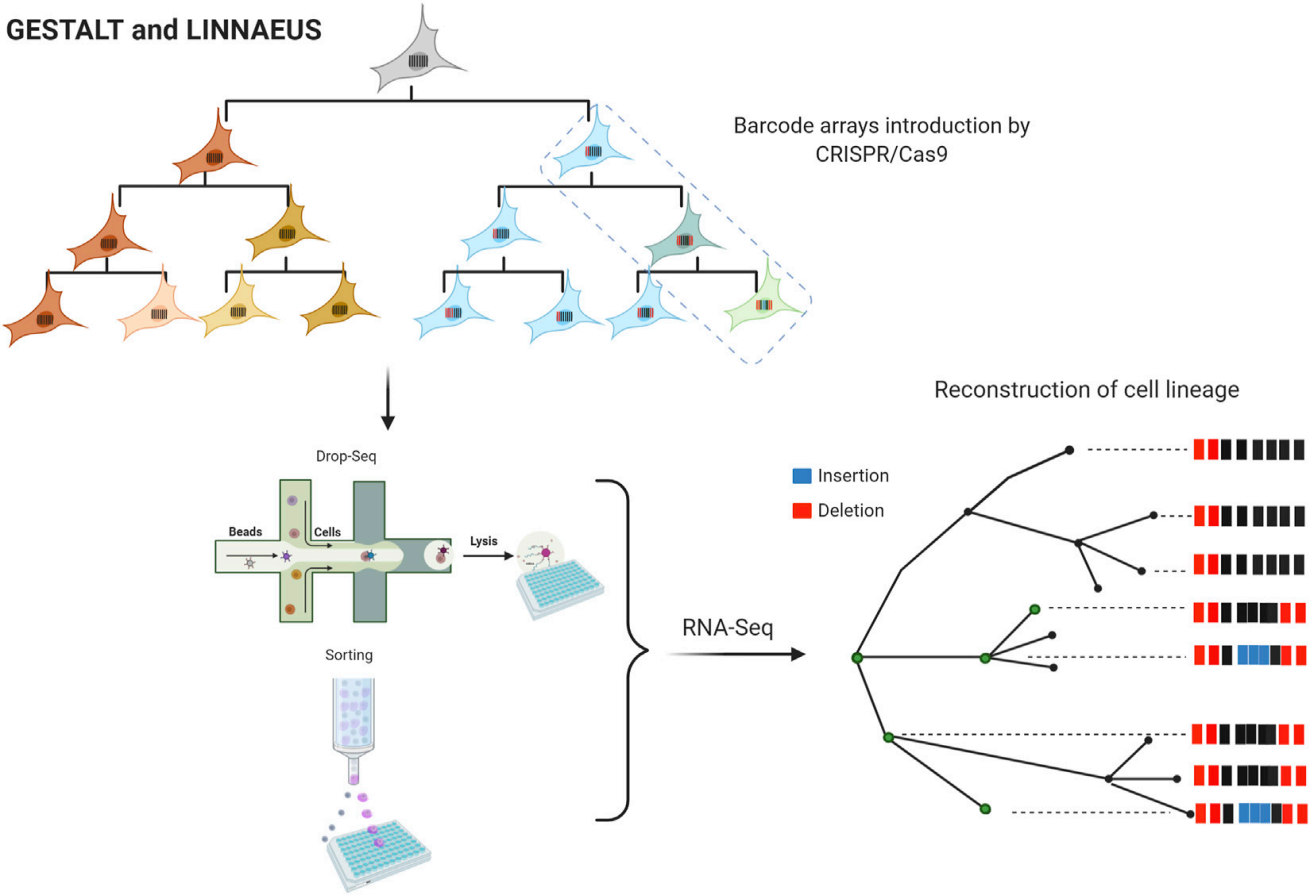
Large-scale maps of cell lineage methods. “Figure created with BioRender.com.” Genome editing of synthetic target arrays for lineage tracing (GESTALT) and LINeage tracing by Nuclease-Activated Editing of Ubiquitous Sequences (LINNAEUS). Barcode arrays are designed as INDELs arrays of different CRISPR/Cas9 target sites and are injected into the embryos at the 1-cell stage. After numerous rounds of cell divisions, the edited targets are accumulated. The single-cell analysis is performed in cells expressing a fluorescence marker by printed droplets microfluidics or cell sorting. RNA-Seq data are analyzed for lineage tree reconstruction identifying shared barcodes.

Meanwhile, cellular barcoding strategies and single-cell sequencing have led to the development of new methodologies for lineage tracing. GESTALT and LINNAEUS are two other emerging methods with the potential to generate large-scale cell lineage maps. Using these methods is possible to introduce barcode arrays that can be traced in future generations using multiple CRISPR/Cas target sites (Figure 3) (McKenna et al., 2016). These barcodes consist of combinations of insertions and deletions (INDELs) generated by CRISPR/Cas9 and are designed to have multiple targets in the genome. After numerous rounds of cell divisions, there is an accumulation of edited targets. It is assumed that targets are independent of each other, which allows lineage tracing; shared barcodes reconstruct cell lineage trees. LINNAEUS and GESTALT methods employ fluorescence intensity of GFP or RFP for INDELs detection; however, LINNAEUS designs are more limited in comparison to GESTALT designs since their arrays are performed in the 3′UTR of GFP or RFP, facilitating the simultaneous detection of multiple targets (Chen et al., 2022). In the long term, all these new approaches will generate invaluable information, representing new challenges due to the amount of data to process and analyze. In other words, the evolution of informatics platforms for understanding single-cell CRISPR screening data is also being considered; MUSIC, scMAGeCK, and SCEPTRE are clear examples of these advances in data analysis (Duan et al., 2019; Yang et al., 2020; Barry et al., 2021; Watson et al., 2021).

### Glial cell’s potential applications in regenerative medicine

In regenerative medicine, constructing cell lineage trees could enhance the therapeutic approaches for converting glial cells into functional neurons, which could benefit a wide range of medicinal purposes. Recently, Zhou et al. (2020) reported an efficient conversion of Müller glia into retinal ganglion cells (RGCs) by downregulation of the polypyrimidine tract-binding protein 1 (Ptbp1), employing an *in vivo* viral delivery and the CRISPR-Cas13d (CasRx), in this research was proven induction of neurons with dopaminergic features in the striatum that contrasted motor defects in a PD mouse model (Zhou et al., 2020). Ptbp1encodes an RNA binding protein whose depletion is sufficient to convert cultured mouse fibroblasts and N2a cells into functional neurons (Xue et al., 2013; Qian et al., 2020). More recently, the conversion of midbrain astrocytes to dopaminergic neurons with the ability to reconstruct the nigrostriatal circuit in an induced mouse PD model has been demonstrated (Qian et al., 2020). These findings further incentivize the potential applications of glial cells as a source of neurons to replace those that lose their function in neurodegenerative diseases.

The use of technologies involving CRISPR/Cas would not just enable the elucidation of the current gaps in glial cells. Still, it would also be a great way to generate fast and reliable models for glial cell studies, as an existing method that allowed to reduce the differentiating time of hPSCs to astrocytes from 3–6 months to generate functional astrocytes from hESCs and hiPSCs in 4–7 weeks, meaning NF1A and SOX9 dispensable at the early stage of neural differentiation (Li et al., 2018)**.** Besides, iPSC-based disease models are a powerful tool for understanding neurodegenerative disorders in a relevant genetic and cellular context. Recently, Guan et al. (2022) reported a promising iPSC generation method with small molecules. Chemical reprogramming has advantages compared to existing reprogramming methods: it is non-integrative to the genome, controllable, and easy to optimize, standardize and manufacture. In addition, differentiation employing small molecules has shown better epigenome reprogramming which is essential for reliable iPSC-based models (Meneghini et al., 2021; Guan et al., 2022).

### Perspectives and remarks

Although the study of glial cells has been delayed by a lack of interest in the past, the use of new biotechnological tools, such as those employing CRISPR/Cas, will decrease the existing knowledge gap in the glial cells. The CRISPR/Cas enhancements and their CRISPR screening platforms along the large-scale maps of cell lineage methods make new genomic sites that were previously inaccessible for genome engineering to study genes of interest in bulk; in addition, emerging Cas proteins could be used in the all-in-one delivery method, which is crucial for success *in vivo* delivery experiments. However, it is essential to consider the limitations of using this technology, which is still improving. For example, introducing specific INDELs tends to be less efficient than KO experiments. On the other hand, the outcomes after genome edition can vary in differentiated neural tissue since the nonhomologous end-joining (NHEJ) DNA repair is the most common in this type of cell, and the CRISPR/Cas system has been optimized in dividing cell lines that generally use the HDR repair. Additionally, the NHEJ pathway is more active and error-prone, facilitating frame-shift mutations in the coding sequence. Although new approaches have been developed such as the strategy “HiTi” (homology-independent targeted integration) to improve DNA knock-in in dividing and nondividing cells (Vesikansa, 2018; Meneghini et al., 2021).

Even though CRISPR specificity has improved over time, it is still a critical concern for clinical applications. On the other hand, it has been reported toxicity after an induced double-strand break (DSB), and there is no information about it in neurons and glial cells. In addition, the complex and diverse architecture of the brain limits accesses to the CRISPR/Cas system (Vesikansa, 2018; Meneghini et al., 2021).

Next-generation sequencing technologies have led to the discovery of novel genes associated with the multiple functions of glial cells as well as their role in the appearance of neurodegenerative diseases without forgetting genes of paramount interest that have been somehow overlooked or not sufficiently investigated; the use of pooled screening, single-cell CRISPR screening, lineage tracing, and transcriptome profiling are powerful strategies to facilitate gene function evaluation to define and manipulate specific subsets of neurons and glial cells, and thus find critical unanswered questions. However, there are some challenges to a successful CRISPR screen study; for example, the appropriate screen design as well as the biological model selection, optimization of the delivery of Cas protein, and the gRNAs, and noise associated or not associated with CRISPR screens (Bock et al., 2022). The use of these technologies is vital to expand current knowledge and go beyond one of the functions most described by microglia, namely, synaptic pruning during development and synaptic modulation, and begin to add missing pieces when it comes to functions, cellular origins, differentiation time, morphology, along with the discovery of new cell types involved in embryogenesis and the CNS.
